# *Anaerolineaceae* and *Methanosaeta* turned to be the dominant microorganisms in alkanes-dependent methanogenic culture after long-term of incubation

**DOI:** 10.1186/s13568-015-0117-4

**Published:** 2015-06-18

**Authors:** Bo Liang, Li-Ying Wang, Serge Maurice Mbadinga, Jin-Feng Liu, Shi-Zhong Yang, Ji-Dong Gu, Bo-Zhong Mu

**Affiliations:** State Key Laboratory of Bioreactor Engineering and Institute of Applied Chemistry, East China University of Science and Technology, Shanghai, People’s Republic of China; School of Biological Sciences, The University of Hong Kong, Pokfulam Road, Hong Kong, People’s Republic of China; Shanghai Collaborative Innovation Center for Biomanufacturing Technology, Shanghai, 200237 People’s Republic of China

**Keywords:** Alkanes degradation, *Anaerolineaceae*, *Methanosaeta*, 16S rRNA gene, Methanogenesis, Microbial community

## Abstract

The methanogenic alkanes-degrading enrichment culture which had been incubated for over 1,300 days amended with *n*-alkanes (C_15_–C_20_) was investigated through clone libraries of bacteria, archaea and *assA*, *mcrA* functional genes. These enrichment cultures were obtained from oily sludge after an initial incubation of the oily sludge without any carbon source and then an enrichment transfer with *n*-alkanes (C_15_–C_20_) for acclimation. Activation of alkanes, methane precursor generation and methanogenic pathways are considered as three pivotal stages for the continuous methanogenesis from degradation of alkanes. The presence of functional genes encoding the alkylsuccinate synthase α-subunit indicated that fumarate addition is most likely the one of initial activation step for degradation of *n*-alkanes. Degradation intermediates of *n*-alkanes were octadecanoate, hexadecanoate, butyrate, isobutyrate, acetate and propionate, which could provide the appropriate substrates for acetate formation. Both methyl coenzyme M reductase gene and 16S rRNA gene analysis showed that microorganisms of *Methanoseata* were the most dominant methanogens, capable of using acetate as the electron donor to produce methane. Bacterial clone libraries showed organisms of *Anaerolineaceae* (within the phylum of *Chloroflexi*) were predominant (45.5%), indicating syntrophically cooperation with *Methanosaeta* archaea was likely involved in the process of methanogenic degradation of alkanes. Alkanes may initially be activated via fumarate addition and degraded to fatty acids, then converted to acetate, which was further converted to methane and carbon dioxide by methanogens.

**Electronic supplementary material:**

The online version of this article (doi:10.1186/s13568-015-0117-4) contains supplementary material, which is available to authorized users.

## Introduction

Methanogenic degradation of alkanes is an important part of microbial enhanced energy recovery (MEER) in which oil in petroleum reservoir may be converted into methane gas. Moreover, it is also an interesting area of research for bioremediation of oil-contaminated environments. Alkanes are quantitatively the most significant components of petroleum hydrocarbons (Head et al. 2010). Comparing to other anaerobic degrading conditions, which use nitrate, nitrite, sulphate, chlorate or ferric iron as the electron acceptor, methanogenic degradation of alkanes has the least Gibbs free energy yield. Zengler et al. (1999) firstly reported an enrichment culture that converted long-chain alkanes (specifically hexadecane) to methane under methanogenic conditions. After that, related researches on methanogenic degradation of alkanes have been reported (Anderson and Lovley 2000; Jones et al. 2008; Mbadinga et al. 2012; Gray et al. 2011; Zhou et al. 2012; Wang et al. 2011, 2012; Li et al. 2012; Aitken et al. 2013; Berdugo-Clavijo and Gieg 2014; Embree et al. 2014; Cheng et al. 2013a; Sherry et al. 2014; Abu Laban et al. 2015).

The methanogenic microbial communities capable of degrading petroleum hydrocarbons are complex consortia containing various fermenting bacteria and methanogens. Gray et al. (2010) collected over 3,000 16S rRNA sequences from published data and concluded that the most frequently detected bacteria in hydrocarbon impacted environments include *Firmicutes*, *γ*-*proteobacteria*, *δ*-*proteobacteria*, *ε*-*proteobacteria*, *β*-*proteobacteria*, *Bacteriodetes*, *Actinobacter*, *α*-*proteobacteria*, *Chloroflexi*, *Thermotogae*, *Nitrospira*, *Spirochaetes*, *Acidobacter*, *Planctomycetes* and OP11. More recently, *Syntrophaceae* (*Smithella*/*Syntrophus*) were found to directly participate in the degradation of alkanes (Bakermans and Madsen 2002; Kasai et al. 2005; Allen et al. 2007; Ramos-Padron et al. 2011; Gray et al. 2011; Cheng et al. 2013b; Embree et al. 2014; Tan et al. 2014a, b). However, not all alkanes-degrading consortia were detected positive for *Syntrophaceae* (*Smithella*/*Syntrophus*) as the dominant bacteria. Li et al. (2012) showed that *Actinobacteria* and *Nitrospirae* were the most abundant bacteria in methanogenic enrichment culture obtained from oilfield production water. An enrichment of microbial community from high-temperature oil field production water contained *Firmicutes*, *Thermodesulfobiaceae*, *Thermotogaceae*, *Nitrospiraceae*, *Dictyoglomaceae*, Candidate division OP8 (Mbadinga et al. 2012). A bacterial consortium isolated from petroleum sludge consisting of *Pseudomonas*, *Achromobacter*, *Bacillus* and *Micromonospora* was also able to degrade *n*-alkanes (Gojgic-Cvijovic et al. 2012). Therefore, other microbes that have the potential of directly degradation of alkanes should not be completely excluded, which is significant for bioremediation in different hydrocarbon contaminated system.

In the present work, we obtained a methanogenic alkanes-degrading enrichment culture with over 1,300 days of incubation as a stable methanogenic alkanes-degrading consortium. We investigated the composition of microbial community and potential functional genes coupling with quantitative PCR analysis. When considering the previous work on enrichment culturing in our laboratory (Wang et al. 2012), the total time of the methanogenic alkanes-degrading enrichment culture was extended for more than 6 years.

## Materials and methods

### Enrichment cultures

In the enrichment culturing process, we transferred about 10 mL (20%) of the inoculum into a 120 mL autoclaved serum bottle containing 40 mL of sterilized basal medium, while the bottle was flushed with pure N_2_ gas after passing through copper filings to remove traces of oxygen. The inocula showing ability of methanogenic degradation of alkanes were taken from the enrichment culture established by Wang et al. (2012). After flushing the serum bottles, they were sealed with butyl rubber stoppers and aluminum crimps (Bellco Glass, Inc., Vineland, NJ, USA).

The basal medium contained (g/L): NaCl, 0.20; MgCl_2_·6H_2_O, 1.20; CaCl_2_·2H_2_O, 0.10; NH_4_Cl, 0.25; KH_2_PO_4_, 0.20; KCl, 1.30; NaHCO_3_, 2.50; rezasurin, 0.0001 and 1 (mL/L) of vitamin stock solution and trace element stock solution as described by Wang et al. (2011). Na_2_S·9H_2_O (0.50 g/L) was used to reduce medium and the final pH of the basal medium was adjusted to 7.2.

Substrates of a mixture of *n*-alkanes (C_15_–C_20_) were supplemented into each 120 mL serum bottle inoculated with the methanogenic alkanes-degrading enrichment culture (three replications). Composition of *n*-alkanes mixture (C_15_–C_20_) were *n*-pentadecane (C15; ≥99%), *n*-hexadecane (C16; ≥99%), *n*-heptadecane (C17; ≥99%), *n*-octadecane (C18; ≥99%), *n*-nonadecane (C19; ≥99%) and *n*-eicosane (C20; ≥99%) (Sigma-Aldrich, Milwaukee, WI, USA). Two sets of control experiments were prepared in the same way as the treatment group: background control group prepared without addition of *n*-alkanes (three replications); autoclaved control group amended with *n*-alkanes but sterilized (three replications).

### Chemical analysis

Gas chromatography (GC) equipped with a 1.5 m stainless-steel column filled with 5 Å carbon molecular sieves and a flame ionization detector (FID) was used to measure the production of methane in the headspace of each serum bottle over time of incubation. Two hundred microlitre of the gas from the headspace were injected into GC with a micro-syringe. The program setting of the GC was as follows: the column temperature was 60°C for 12 min, and then the temperature increased to 200°C at the rate of 15°C/min, at the final temperature of 200°C maintained for 24 min. The injector, TCD and FID temperatures were set at 200°C. The standard calibration curves of methane and hydrogen were made based on relationship between peak area and the respective concentrations (R^2^ = 0.994, *n* = 6).

The residual *n*-alkanes and intermediate metabolites in enrichment cultures of incubation were detected at the end of incubation through GC–MS, an Agilent 6890 GC fitted with a HP-5MS capillary column (30 m × 0.25 mm × 0.25 μm) and 5975 MS in full scan mode (Agilent Technologies, Inc.). For detection of residual *n*-alkanes: 50.0 μL of cetyl chloride as a surrogate standard was added into the serum bottle, then the culture aliquot was extracted with hexane and dried over anhydrous Na_2_SO_4_. Pooled organic layer, transferred to a new vial, and concentrated under a stream of N_2_. The extracts were injected into GC–MS, program setting was initial oven temperature at 120°C for 3 min, and increased at a rate of 8°C/min to 260°C maintained for 10 min. GC peak area of surrogate standard (cetyl chloride) and alkanes ranging from C_15_ to C_20_ were integrated, then obtained the *n*-alkane-to-standard ratio. Finally, the quantity of each *n*-alkane with the peak area ratio was calculated. Intermediate metabolites mainly included long chain fatty acids (LCFAs) and volatile fatty acids (VFAs). Five mL of culture aliquot of the sample were taken respectively; added with ammonia water to raise pH to >12 and dried in an oven at 110°C; esterification at 90°C for 60 min by adding 0.5 mL of 10% butanol/sulfate solution. Then LCFAs and VFAs were extracted with 0.5 mL *n*-hexane and 0.5 mL *n*-dodecane, respectively. The extracts then injected onto the GC–MS. For LCFAs analysis: oven temperature was maintained at 120°C for 3 min, increased at a rate of 8°C/min to 260°C and maintained for 10 min, and for the VFAs analysis: oven temperature was maintained at 60°C for 1 min, increased at a rate of 15°C/min to 130°C.

### DNA extraction and PCR-amplification

We extracted the total genomic DNA after 1,300 days of incubation. Ten millilitre of active enrichment culture samples were taken from the serum bottles (same bottles as for chemical analysis) and centrifuged at 12,000×*g* for 10 min and the settling materials on the bottom were used for genomic DNA extraction by using a commercial extraction kit (Axygen Biosciences, USA) according to its instructions.

Partial 16S rRNA genes of bacteria and archaea were amplified using the bacterial primer 8F/805R (Savage et al. 2010) and archaeal primer A109F/A915R (Nazina et al. 2006; Weisburg et al. 1991), respectively. PCR conditions for bacterial 16S rRNA genes were as follows: 95°C for 5 min, followed by 38 cycles of 95°C for 30 s, 52°C for 45 s, 72°C for 60 s, and a final elongation step at 72°C for 10 min. PCR conditions for archaeal 16S rRNA genes were as follows: 95°C for 5 min, followed by ten cycles of 95°C for 30 s, 63°C for 30 s (decreased by 0.5°C per cycle to 58°C), 72°C for 60 s, after the touchdown 28 additional cycles in which annealing temperature of 58°C were performed and a final elongation step at 72°C for 10 min.

The alkylsuccinate synthetase genes (*assA*) and methyl coenzyme-M reductase genes (*mcrA*) were also amplified. The gene *assA* (alkylsuccinate synthase alpha-subunit) encoding alkylsuccinate synthetase (ASS) was considered as a biomarker for detecting fumarate addition pathway in degradation of alkanes (Callaghan et al. 2010). In recent years several researchers have reported the discovery of *assA* gene in their methanogenic consortia (Zhou et al. 2012; Mbadinga et al. 2012; Li et al. 2012; Cheng et al. 2013c; Wang et al. 2012; Tan et al. 2013; Kolukirik et al. 2011; Berdugo-Clavijo and Gieg 2014; Tan et al. 2014b; Abu Laban et al. 2015). Methyl coenzyme-M reductase gene (*mcrA*) is a key gene in the terminal step of methane-producing pathway, the enzyme catalyzes the production of methane by reducing the methyl group bound to coenzyme-M. This gene could be a biomarker in the detection of methanogens (Thauer 1998). The primers of the functional genes used in this study are given in Additional file 1: Table S1. PCR conditions were as follows: 95°C for 5 min, followed by 10 cycles of 95°C for 30 s, 60°C for 30 s (decreased by 0.5°C per cycle to 55°C), 72°C for 60 s, after the touchdown 30 additional cycles in which annealing temperature of 55°C were performed and a final elongation step at 72°C for 10 min.

### Construction of 16S rRNA genes and *assA*, *mcrA* functional genes libraries

The PCR-amplified and then purified bacterial and archaeal 16S rRNA and *assA*, *mcrA*, functional gene fragments were cloned into *E. coli* using pMD19^®^-T simple vector kit (TaKaRa Bio Inc., Japan) according to the manufacturer instructions. White clones were picked randomly and inoculated into 1 mL of Luria Broth (LB) medium containing ampicillin and incubated at 37°C for 24 h. Primer set M13-47 (5´-CGCCAGGGTTTTCCCAGTCACGAC-3´) and RV-M (5´-GAGCGGATAACAATTTCACACAGG-3´) was used to determine the positive clones. Sequencing of the positive clones was performed on an ABI 377 automated sequencer. Sequences of the respective clone libraries were trimmed to remove vector and primer sequences using MEGA6.0 software (Tamura et al. 2013). Chimeric sequences of 16S rRNA gene were checked and removed by Bellerophon (Huber et al. 2004). Operational taxonomic units (OTUs) were classified using FastGroupII (Yu et al. 2006) with the 97% similarity. The OTUs of *assA*, *mcrA* functional genes were translated using ExPASY translation tool (http://web.expasy.org/translate/). Gene sequences of 16S rRNA and the protein sequences of functional genes were compared to the GenBank Database using BLAST to match the most similar sequences. Phylogenetic and molecular evolutionary analyses were conducted using MEGA6.0 software (Tamura et al. 2013) with neighbor-joining method (Saitou and Nei 1987) and 1,000 bootstrap replicates.

The 16S rRNA gene sequences and *assA*, *mcrA* functional gene sequences from the clone libraries were deposited in GenBank with the accession numbers KJ432869 to KJ432905 and KJ461623 to KJ461653.

### 16S rRNA and functional genes quantitative PCR assay

The copies of 16S rRNA genes of *Archaea*, *Bacteria*, *Methanosarcinaceae*, *Methanosaetaceae* and functional genes of *assA*, *mcrA* were quantified by using SYBR Green I Real-Time PCR (BioRad CFX96 thermocycler, Bio-Rad Laboratories Inc., USA). A ten-fold dilution series of plasmids containing target DNA sequences were chosen as a calibration standard for establishing the standard curve. The primers used in the quantitative PCR are shown in Additional file 1: Table S1. Twenty μL volume of quantitative PCR reactions comprised SYBR Green Realtime PCR Master Mix-Plus (10 μL) (TaKaRa Bio Inc., Japan), Plus Solution 2 μL (TaKaRa Bio Inc., Japan), PCR primers (1 μL of each), sterile water (4 μL), DNA sample (2 μL). The q-PCR conditions were pre-denaturation at 95°C for 3 min, followed by 38 cycles of denaturation at 94°C for 20 s, annealing at specific primer temperature (Additional file 1: Table S1) for 30 s, elongation at 72°C for 60 s.

## Results

### The residual *n*-alkanes, intermediate metabolites and headspace methane generation

**Figure 1:**
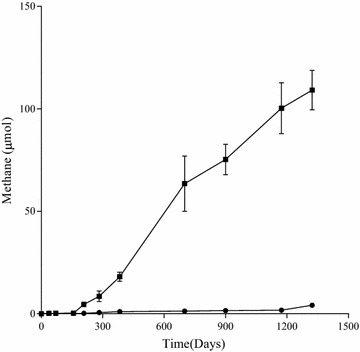
Methane production of the *n*-alkanes degradation consortium under methanogenic conditions. The curve with (*filled square*) means incubated with *n*-alkanes mixture (C_15_–C_20_) as the sole carbon and energy source (three replications) and (*filled circle*) means without any n-alkanes and other carbon sources as the control group (three replications).

In this study, the methanogenic enrichment culture amended with *n*-alkanes had a lag phase of 157 days, after that it began to produce detectable methane and accumulated to a total of 109.2 μmol of methane after 1,323 days. The control group without any carbon source generated 3.9 μmol of methane after 1,323 days of incubation as background level (Figure 1). We have successfully detected the residual *n*-alkanes and intermediate metabolites (LCFAs and VFAs), after more than 1,300 days of incubation under methanogenic conditions. The consumption of *n*-alkanes in active enrichment cultures was obtained by subtracting the substrates remained in the autoclaved controls by the residuals in the active enrichment cultures. The net loss of total *n*-alkanes was 19.85 μmol, by assuming all the consumed *n*-alkanes were used for producing methane, the predicted methane produced should reach 276.03 μmol (Symons and Buswell 1933; Zengler et al. 1999). Considering the detected methane of 109.2 μmol, methane accumulated in headspace accounted for 39.56% of theoretically predicted value. Intermediate metabolites were octadecanoate, hexadecanoate, butyrate, isobutyrate, acetate and propionate.

### Phylogenetic analyses of bacteria

**Figure 2:**
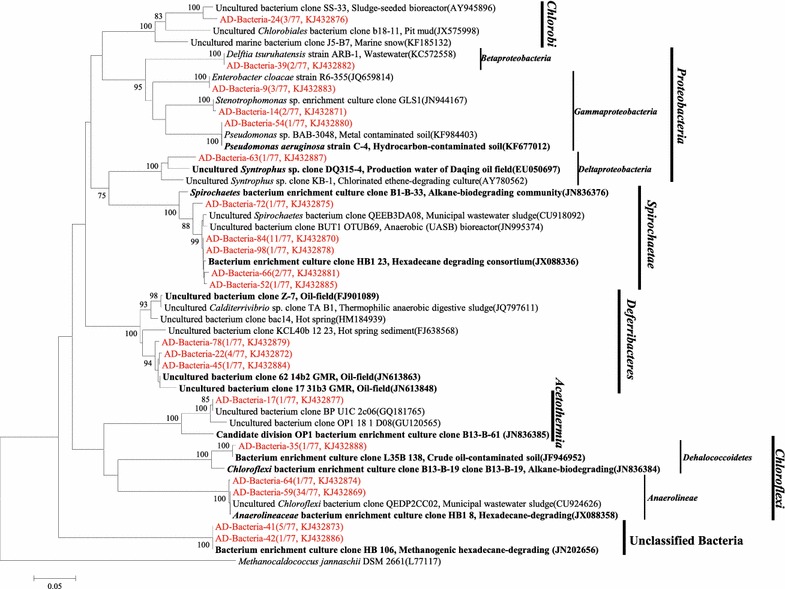
Phylogenetic tree of bacterial 16S rRNA gene sequences from methanogenic alkanes-degrading enrichment culture (in *red*) and rooted with outgroup sequence from *Methanocaldococcus jannaschii* DSM 2661 (L77117). The OTUs are shown with clone names and accession numbers. Sequences from oil or hydrocarbon-impacted environments are in *black bold*. Values below 75% are not shown. The topology of the tree was obtained by the neighbor-joining method. Bootstrap values (*n* = 1,000 replicates).

For the phylogenetic analysis of bacteria, more than 100 clones were picked and sequenced. In total 77 valid sequences after removing the vector and chimeric sequences were obtained. These 77 valid sequences were analyzed and classified into 20 OTUs by FastGroupII with the 97% similarity (Figure 2). The OTU (AD-Bacteria-59) contains the most sequences (34 out of 77 valid sequences followed by OTU (AD-Bacteria-84) with 11 sequences. OTU (AD-Bacteria-59) belongs to *Anaerolineae* within the phylum *Chloroflexi* and OTU (AD-Bacteria-84) belongs to *Spirochaetae*. Phylogenetic analysis reveals that these 20 OTUs affiliated with *β*-, *γ*-, *δ*-*Proteobacteria*, *Spirochaetae*, *Deferribacteres*, *Chlorobi* and C*hloroflexi* (Figure 2). Among these bacteria, C*hloroflexi* have the highest number of clones (46.7%), followed by *Spirochaetae* (21.0%), and the remaining of 32.3% belongs to the phylum of *Proteobacteria*, *Deferribacteres*, *Chlorobi*, *Acetothermia* and Unclassified Bacteria. Sequences with high similarity from oil or hydrocarbon-impacted environments are highlighted in bold and black (Figure 2).

### Phylogenetic analyses of Archaea based on 16S rRNA and *mcrA* genes

**Figure 3:**
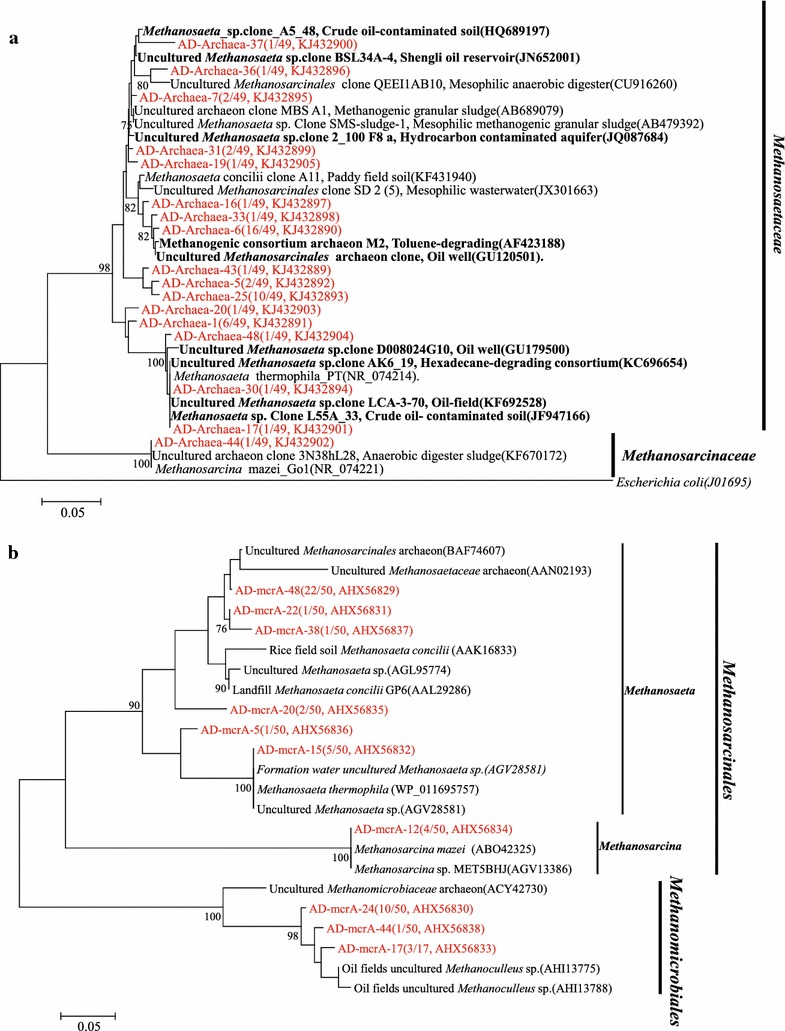
Phylogenetic tree of archaeal 16S rRNA gene sequences from methanogenic alkanes-degrading enrichment culture (in *red*) and rooted with outgroup sequence from *Escherichia coli* (J01695). The OTUs are shown with clone names and accession numbers. Sequences from oil or hydrocarbon-impacted environments are in *black bold*. The topology of the tree was obtained by the neighbor-joining method. Bootstrap values (*n* = 1,000 replicates), the values below 75% are not shown (**a**); phylogenetic tree of deduced amino acid sequences of methyl coenzyme-M reductase genes (*mcrA*) from methanogenic alkanes-degrading enrichment culture (in *red*). Topology of the tree was obtained by the neighbor-joining method. The evolutionary distances were computed using the Poisson correction method. Bootstrap values (*n* = 1,000 replicates), values below 75% are not shown (**b**).

In total 49 valid sequences were obtained from 60 archaeal clones after vector and chimeric checking, resulting in 17 OTUs by FastGroupII with the 97% similarity (Figure 3a). All of the 17 OTUs belong to *Methanosarcinales*, in which *Methanosaeta* accounts for 98% and *Methanosarcina* only accounts for the remaining 2%. Obviously, *Methanosaeta* was the dominant archaea by 16S rRNA gene clone libraries analysis. Sequences with high similarity from oil or hydrocarbon-impacted environments are highlighted in bold and black (Figure 3a).

For *mcrA* genes clone library analysis, 50 clones were picked randomly for sequencing and resulted ten OTUs by FastGroupII with the 97% similarity (Figure 3b). OTU of AD-mcrA-48 contains the most clones. A 72.0% of the clones belongs to the order of *Methanosarcinales* which covers the genus of *Methanosaeta* (64%) and *Methanosarcina* (8%), and the remaining 28.0% clones belongs to the order of *Methanomicrobiales* which was not detected in the archaeal 16S rRNA gene clone library.

### Phylogenetic analysis of *assA* functional gene

**Figure 4:**
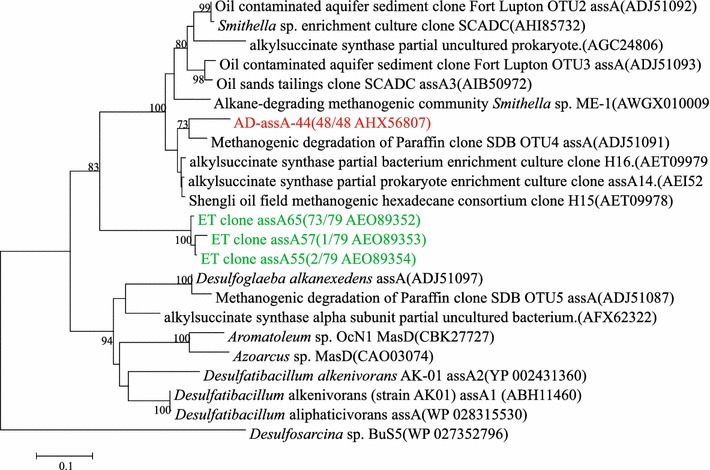
Phylogenetic tree of deduced amino acid sequences of alkylsuccinate synthetase genes (*assA*) genes from methanogenic alkanes-degrading enrichment culture (in *red*). Clone sequences with* green* were detected in the stage III by Wang et al. (2012). The topology of the tree was obtained by the maximum likelihood method. Bootstrap values (*n* = 1,000 replicates), values below 70% are not shown.

The primer set *assA*2F/*assA*2R was chosen for PCR amplification (Additional file 1: Table S1). In total 48 sequences were analyzed through FastGroupII with the 97% similarity and only one OTU was found (Figure 4). Phylogenetic analysis of the deduced amino acid revealed that this OTU was 90% similar to gene sequence (AET09978) from Shengli oil field (Cheng et al. 2013c) and closely related to the strain *Smithella* sp. The primer set 1432F and *ass*/*bss*R (Callaghan et al. 2010) used by Wang et al. (2012) did not generate any positive products.

### Quantitative PCR

The quantitative PCR of 16S rRNA genes of *Archaea*, *Bacteria*, *Methanosarcinaceae*, *Methanosaetaceae* and functional genes *assA*, *mcrA* from methanogenic alkanes-degrading enrichment culture consortia were performed in BioRad CFX96 thermocycler. In the q-PCR reactions the efficiency was between 93.1 and 120%, R^2^ values were above 0.958 (triplicate samples). The log gene copies of *Archaea*, *Bacteria*, *Methanosarcinaceae*, *Methanosaetaceae*, *assA* and *mcrA* per milliliter of culture aliquot were 7.62 ± 0.38, 6.36 ± 0.084, 4.85 ± 0.05, 6.43 ± 0.33, 3.93 ± 0.03 and 5.20 ± 0.09, respectively.

## Discussion

The microbial community of anaerobic methanogenic degradation of alkanes has been incubated for over 6 years in four stages: original oily sludge sample from Shanghai Oil Refinery (Stage I); initial enrichment culture of oily sludge sample without any additional organic carbon source incubated for more than 500 days (Stage II); the first methanogenic enrichment transfer incubation from “Stage II” amended with *n*-alkanes as the sole carbon source incubated for another 500 days (Stage III); the second enrichment transfer incubation from “Stage III” incubated for over 1,300 days (Stage IV). Investigation of stage I, II, III had been accomplished previously by Wang et al. (2012), the stage IV was conducted in the present research. A total of 109.2 μmol of methane were detected after 1,300 days of incubation, and the gas reached nearly 50 μmol after 500 days, this was more than recorded previously in the first transfer of culture after 500 days (18 μmol). Obviously, a more stable and efficient consortium for methanogenesis from alkane in the second enrichment transfer culture was obtained. The residual *n*-alkanes and presence of the intermediates including octadecanoate, hexadecanoate, butyrate, isobutyrate, acetate and propionate, suggested that the methanogenic alkanes-degrading process is still active in process.

### *Anaerolineaceae* turned to be the dominant bacteria after long-term incubation under alkanes-dependent methanogenic conditions

**Figure 5:**
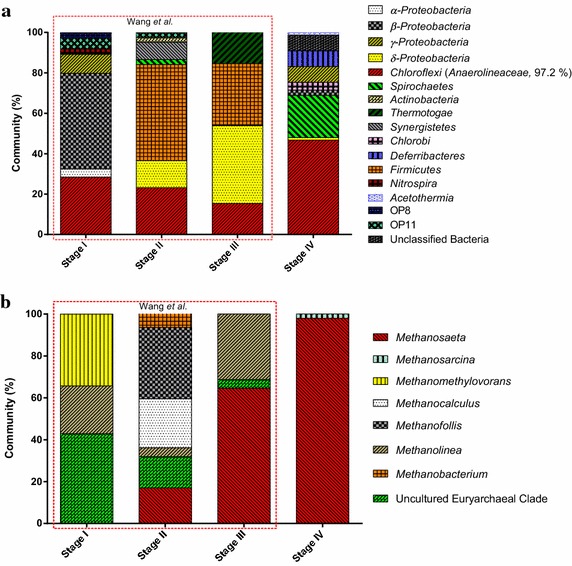
The microbial community varied with the time of incubation from the analysis of bacteria and archaea 16S rRNA gene clone libraries. Relative proportion of bacteria lineages (**a**) and archaea lineages (**b**) in the four stages during the methanogenic alkanes-degrading enrichment culture. “Stage I” represents the original oily sludge sample from Shanghai Oil Refinery; “Stage II” represents the initial enrichment culture of oily sludge sample without any additional carbon source and incubated for more than 500 days; “Stage III” represents methanogenic enrichment transfer incubation from “Stage II” amended with *n*-alkanes as the sole carbon source for another 500 days, for details see Wang et al. (2012). “Stage IV” represents the second transfer incubation from “Stage III” for over 1,300 days.

Bacterial community varied over the time of incubation (Figure 5a). The most obvious change in the bacterial community was that the phylum of *Chloroflexi* (vast majority of the family *Anaerolineaceae*) became the most dominant bacteria after 6 years of incubation. Comparing to the first and second transfer incubation (Stage III and Stage IV), the dominant consortium of bacteria had shifted from *δ*-*Proteobacteria* (38.5%) and *Firmicutes* (30.8%) to the phylum of *Chloroflexi* (46.7%) and *Spirochaetae* (21.0%). The clones affiliated with *δ*-*Proteobacteria* decreased to 1.3% while *Firmicutes* clones were not detectable any more. After 500 days of the initial enrichment culture, relative abundance of *Chloroflexi* remains steady from 28.4% (Stage I) to 23.2% (Stage II). In Stage III after first transfer incubation, abundance of *Chloroflexi* dropped gently to 15.4%. For this slight drop in Stage III, when *n*-alkanes were amended as the sole carbon source, *Chloroflexi* might face a situation where some growth factors may be depleted leading to a longer lag phase for *Chloroflexi*. After the second transfer incubation of 1,300 days (Stage IV) when acclimated into the environmental conditions, *Chloroflexi* increased sharply from the initial amount of 15.4–46.7%.

Numerous studies have demonstrated that *Syntrophaceae* (*Smithella*/*Syntrophus*) play a key role in hydrocarbon contained system and are responsible for alkane activation and LCFA oxidation (Bakermans and Madsen 2002; Kasai et al. 2005; Allen et al. 2007; Ramos-Padron et al. 2011; Gray et al. 2011; Cheng et al. 2013a; Embree et al. 2014; Tan et al. 2014a, b; Zengler et al. 1999). Whereas, only one sequence (KJ432887) affiliated with *Syntrophaceae*, accounts for 1.3% of the total bacterial sequences, was detected in our consortium. Interestingly, *Anaerolineaceae* turned to be the most predominant bacteria (nearly a half), which means not only *Syntrophus* or *Smithella*-like bacteria can contribute for the methanogenic degradation of alkanes, *Anaerolineaceae* are also worth to be concerned. *Anaerolineaceae* comprise obligate anaerobes, as a majority in our alkanes-degrading consortium is not a surprise, they have occurred in many oil and hydrocarbon environments. In order to illustrate that *Anaerolineaceae* played an important role in hydrocarbon degradation, a survey of *Anaerolineaceae* associated with oil and hydrocarbon environment are summarized in Table 1. Ficker et al. (1999) firstly reported that *Chloroflexi* was related to toluene degradation in a 10-years period toluene-degrading methanogenic consortium. Sutton et al. (2013) found that *Anaerolineaceae* may be associated with the anaerobic degradation of oil-related compounds, this bacterial lineage was also reported as the most frequently encountered bacteria taxon in anaerobic *n*-alkane degradation (Sherry et al. 2013). Savage et al. (2010) detected that *Anaerolineaceae* as one of the community members in metabolism of low-molecular-weight alkanes (*n*-propane and *n*-pentane) under mesophilic sulfate-reducing conditions, which showed the potential of hydrocarbon degradation for these lineage organisms. Several researchers found that the class of *Anaerolineae* has the traits of filamentous morphology, heterotrophic and low growth rate in anaerobic condition (Sekiguchi et al. 2001). The formation of sludge granules by *Anaerolineae* was commonly detected and considered important for the settleability in upflow anaerobic sludge blanket system (Yamada and Sekiguchi 2009). This filamentous morphology may facilitate the degradation of hydrocarbons by *Anaerolineaceae* in cooperation with other microorganisms. Because filamentous morphology is conducive to the granulation (Guiot et al. 1992; Yamada et al. 2005), an effective way for aggregation of microbes and substrates. Community genomic analyses indicated that most members in the phylum of *Chloroflexi* are anaerobic acetogenic microbes, which also have the genomes of complete Wood–Ljungdahl pathway and β-oxidation of saturated fatty acids (Hug et al. 2013). Thus, we can infer that *Chloroflexi* (more specifically, *Anaerolineaceae*) have the ability of providing organic acid (such as acetate) to other microorganisms like acetoclastic methanogens.

**Table 1:** A survey of oil and hydrocarbon associated *Anaerolineaceae*

References	Accession number	Clone/strain	Isolation source	Region	Percentage^a^
This study	KJ432869	AD_Bacteria_59	Methanogenic *n*-alkanes-degrading consortium from oily sludge enrichment cultures for over 1,300 days	China	100
Sun and Cupples (2012)	JN806351	DSS-20	Toluene-degrading microbial communities	USA	100
	JN806343	DSS-12	Toluene-degrading microbial communities	USA	99
Yan et al. (2006)	DQ080186	B45	The reductive dechlorination of 2,3,4,5-tetrachlorobiphenyl in three different sediment cultures	USA	96
Winderl et al. (2008)	EU266919	D25_46	Toluene degraders in tar-oil contaminated aquifer sediments	Germany	96
	EU266901	D25_25	Toluene degraders in tar-oil contaminated aquifer sediments	Germany	96
Tischer et al. (2013)	JQ087241	Clone 1_76_3_b	Push core sediment sample from the vadose zone of a hydrocarbon contaminated aquifer	Germany	95
Li et al. (Genbank)^b^	KJ730067	Clone B199	Biogas digester sediment	China	94
Sherry et al. (2013)	JQ033882	SRO176E01	Anaerobic biodegradation of crude oil under sulphate-reducing conditions in 176 days incubation period	UK	93
	JQ033889	SRO302B05	Anaerobic biodegradation of crude oil under sulphate-reducing conditions in 302 days incubation period	UK	93
Lienen et al. (Genbank)^b^	KF147579	Clone 7932270	Mesophilic anaerobic digester in full-scale biogas plant	Germany	93
Gieg et al. (2008)	EU037975	lg1e04	Bioenergy production via microbial conversion of residual oil to natural gas	USA	93
Dojka et al. (1998)	AF050570	WCHB1-57	Hydrocarbon and chlorinated-solvent-contaminated aquifer	USA	93
Gray et al. (2011)	GU996561	MO302A7	Crude oil degrading methanogenic microcosms, 302 days	UK	92
Gieg et al. (2008)	EU037963	E449-5	Bioenergy production via microbial conversion of residual oil to natural gas	USA	91
Gray et al. (2011)	GU996562	MO302C11	Crude oil degrading methanogenic microcosms, 302 days	UK	91
Penner et al. (Genbank)^b^	EU522649	Ctrl1-8D	Hydrocarbon-degrading methanogenic microbial consortia from oil sands tailings	Canada	91
Lv et al. (Genbank)^b^	KJ468504	Clone B16-24	Palmitate degradation microcosm in oil field	China	90
Yamada et al. (2007)	NR041354	GOMI-1	Methanogenic propionate-degrading consortia in thermophilic digester sludge	Japan	90
	NR041355	Strain KOME-1	Methanogenic propionate-degrading consortia in thermophilic digester sludge	Japan	90
Dojka et al. (1998)	AF050569	WCHB1-31	Hydrocarbon and chlorinated-solvent-contaminated aquifer	USA	90
Allen et al. (2007)	DQ663957	5C38	Petroleum-contaminated sediments	USA	90
Abu Laban et al. (2015)	KJ635758	IsoM-30	Biodegradation of C_7_ and C_8_ iso-alkanes under methanogenic conditions	Canada	90
	KJ635796	IsoM-108	Biodegradation of C_7_ and C_8_ iso-alkanes under methanogenic conditions	Canada	90
Herrmann et al. (2008)	EF417532	BAC19-1	In situ microcosms in a monitoring well of an anoxic benzene-contaminated aquifer	Germany	89
Schlötelburg et al. (2000)	AJ249111	SHA-53	Bacteria of an anaerobic 1,2-dichloropropane-dechlorinating mixed culture	Germany	89
Winderl et al. (2008)	EU266859	D15_19	Toluene degraders in tar-oil contaminated aquifer sediments	Germany	89
	EU266865	D15_26	Toluene degraders in tar-oil contaminated aquifer sediments	Germany	88
Abu Laban et al. (2015)	KJ635769	IsoM-41	Biodegradation of C_7_ and C_8_ iso-alkanes under methanogenic conditions	Canada	88
Kuppardt et al. (Genbank)^b^	KF443371	Zz9-00_C6	Toluene-degrading consortia	Germany	87
Savage et al. (2010)	GU211123	ZodOTU50-C07	Biodegradation of low-molecular-weight alkanes under mesophilic, sulfate-reducing conditions	USA	87
Riviere et al. (2009)	CU922908	QEDR2BG03	Mesophilic anaerobic digester which treats municipal wastewater sludge	France	87
Fang (Genbank)^b^	JQ772434	420DB-7	High-temperature oil field production fluids incubated for 420 days	China	86
Cheng and Lu (Genbank)^b^	JX088360	HB1_18	Methanogenic hexadecane-degrading consortium at different incubation temperatures enriched with crude oil-contaminated soil	China	86
	JX088361	LB1_11	Methanogenic hexadecane-degrading consortium at different incubation temperatures enriched with crude oil-contaminated soil	China	86
Savage et al. (2010)	GU211124	ZodOTU21-C08	Biodegradation of low-molecular-weight alkanes under mesophilic sulfate-reducing conditions	USA	85
Cheng and Lu (Genbank)^b^	JF946983	Clone L35B_120	Methanogenic hexadecane-degrading consortium at different incubation temperatures enriched with crude oil-contaminated soil	China	84
	JF946983	Clone L35B_120	Methanogenic hexadecane-degrading consortium at different incubation temperatures enriched with crude oil-contaminated soil	China	84
Wang et al. (Genbank)^b^	JN038290	EK_Ca689	Petroleum-contaminated soil	China	81
Ficker et al. (1999)	AF423186	Eub-6	Toluene-degrading methanogenic consortium	Canada	81
Winderl et al. (2008)	EU266855	D15_15	Toluene degraders in tar-oil contaminated aquifer sediments	Germany	80
Militon et al. (2010)	AM935699	AMCH2	Pilot-scale bioremediation process of a hydrocarbon-contaminated soil	France	77

Family level assignment based on RDP taxonomic classification.

^a^Sequence identity with KJ432869 (this study) determined by BLAST.

^b^Unpublished.

Since the *Spirochaetae* was the predominant bacteria after *Chloroflexi*, it must play an important role during the degradation of alkanes. Some researchers had shown *Spirochaeta* as a participator in hydrocarbon degradation (Paissé et al. 2008; Berdugo-Clavijo et al. 2012; Hirschler-Réa et al. 2012; Kobayashi et al. 2012), besides, a mesophilic strictly anaerobic strain *Spirochaetas maragdinae* sp. has been isolated from oil field (Magot et al. 1997). Although *Deferribacteres* was detected at a low level (7.8%), it should not be ignored. *Deferribacter thermophilus*, which could utilize complex organic compounds and small molecules substrates like hydrogen and acetate as electron donors, has been isolated from a submarine oil reservoir (Greene et al. 1997). This kind of microorganism was also detected in methanogenic enrichment culture from oil reservoir in our laboratory before (Wang et al. 2011).

### *Methanosaeta* turned to be the dominant *Archaea* after long-term incubation under alkanes-dependent methanogenic conditions

After additional 1,300 days of incubation amended with *n*-alkanes, *Methanosaeta* were still the dominant organisms, and the proportion increased from 64.6 to 98.0% (Figure 5b). 16S rRNA gene clone libraries showed that *Methanosaeta* was 50 times more than *Methanosarcina*, which was consistent with q-PCR data f (log gene copies was 6.43 ± 0.33 and 4.85 ± 0.05, respectively). Both *Methanosaetaceae* and *Methanosarcinaceae* were acetoclastic methanogens which take part in converting acetate into methane and carbon dioxide directly (Ferry 1993), *Methanosarcinaceae* could also generate methane by utilizing methyl, hydrogen, carbon dioxide and formic acid. Detection of *mcrA* functional gene suggests *Methanosaeta* (64%) was still the dominant organism. *mcrA* gene affiliated with *Methanomicrobiales* was detected but not in 16S rRNA gene clone library. This may be due to the too little of *Methanomicrobiales* in the system. In addition to that, since the acetogens of *Anaerolineaceae* detected as the most dominant bacteria, plenty of acetate should have generated during the intermediate metabolites. However, the acetate was detected in trace amount. *Methanosaeta* could have consumed acetate once it was generated and this may lead to the minimal residual of acetate. All of the evidence presented above shows that aceticlastic methanogenesis becomes the most predominant methanogenic pathway.

### Possible metabolic pathway of methanogenic alkane’s degradation

For the initial activation of alkanes, three mechanisms are considered to be involved, they are subterminal addition to fumarate, intra-hydroxylation and anaerobic hydroxylation followed by carboxylation (Callaghan 2013). Among them, fumarate addition is the most prevalent and best characterized mechanism in anaerobic hydrocarbon degradation at present (Aitken et al. 2013). The presence of *assA* functional gene suggests that fumarate addition may be involved in the initial activation of alkanes in this methanogenic alkanes-degrading culture but alternative activation mechanisms still cannot be excluded. Assuming that the *assA* gene detected in our methanogenic culture are from members of the *Proteobacteria* and since the *Proteobacteria* represented about 11.7% of the bacterial sequences analyzed, our assumption is consistent with the low copy number of the *assA* gene in the culture. On the other hand, it is possible and even likely that the genes originated from other organisms within the consortium. However, this remains to be elucidated.

This methanogenic alkanes-degrading enrichment culture contains a complicated consortium including bacteria and archaea, which utilize *n*-alkanes as the sole carbon source. *Anaerolineaceae* as the major bacteria would have played an important role in the process of fermentation and oxidation of alkanes, and generated small molecules like formate, acetate, hydrogen, carbon dioxide. Finally, *Methanosaeta* ultimately metabolized acetate to methane and carbon dioxide through acetoclastic pathway.

## Additional files

Additional file 1: Table S1. Characteristics of primers for quantitative PCR.
