# Supplementary material for: Anaerolineaceae and Methanosaeta turned to be the dominant microorganisms in alkanes-dependent methanogenic culture after long-term of incubation
Source: AMB Express. 2015 Jun 18;5:37. doi: 10.1186/s13568-015-0117-4 (PMC4469597; doi:10.1186/s13568-015-0117-4)
Supplement: Additional file 1: Table S1. — Characteristics of primers for quantitative PCR. [file 13568_2015_117_MOESM1_ESM.doc]

**Supplementary Information**

**AMB Express**

***Anaerolineaceae* and *Methanosaeta* turned to be the dominant microorganisms in alkanes-dependent methanogenic culture after long-term of incubation**

Bo Liang^1^, Li-Ying Wang^1^, Serge Maurice Mbadinga^1, 3^, Jin-Feng Liu^1, 3^, Shi-Zhong Yang^1, 3^, Ji-Dong Gu^2^, Bo-Zhong Mu^1, 3,*^

^1^State Key Laboratory of Bioreactor Engineering and Institute of Applied Chemistry, East China University of Science and Technology, Shanghai, P.R. China

^2^School of Biological Sciences, The University of Hong Kong, Pokfulam Road, Hong Kong, P.R. China

^3^Shanghai Collaborative Innovation Center for Biomanufacturing Technology,

Shanghai 200237, P.R. China*Correspondence: Bo-Zhong Mu

E-mail: bzmu@ecust.edu.cn

Phone: +86 21 64252063; Fax: +86 21 64252485

**Table S1**

**Table S1. Characteristics of primers for quantitative PCR**

| **Target group** | **Primer name** | **Sequence (5’→3’)** | **Amplicon length** | **Annealing temperature** | **References** |
| --- | --- | --- | --- | --- | --- |
| ***Archaea*** | ARC787F | ATTAGATACC CSBGTAGTCC | 273 (bp) | 60 °C | (Yu et al. 2005) |
|  | ARC1059R | GCCATGCACCWCCTCT |  |  |  |
| ***Bacteria*** | BAC338F | ACTCCTACGGGAGGCAG | 468 (bp) | 57 °C | (Yu et al. 2005) |
|  | BAC805R | GACTACCAGGGTATCTAATCC |  |  |  |
| ***Methanosarcinales*** | MSL812F | GTAAACGATRYTCGCTAGGT | 354 (bp) | 60 °C | (Yu et al. 2005) |
|  | MSL1159R | GGTCCCCACAGWGTACC |  |  |  |
| ***Methanosarcinaceae*** | Msc380F | GAAACCGYGATAAGGGGA | 448 (bp) | 50 °C | (Yu et al. 2005) |
|  | Msc828R | TAGCGARCATCGTTTACG |  |  |  |
| ***Methanosaetaceae*** | Mst702F | TAATCCTYGARGGACCACCA | 164 (bp) | 50 °C | (Yu et al. 2005) |
|  | Mst862R | CCTACGGCACCRACMAC |  |  |  |
| ***assA*** | assA2F | YATGWACTGGCACGGMCA | 443 (bp) | 55 °C | (Aitken et al. 2013) |
|  | assA2R | GCRTTTTCMACCCAKGTA |  |  |  |
| ***mcrA*** | MLF | GGTGGTGTMGGATTCACACARTAYGCWACAGC | 440 (bp) | 58 °C | (Luton et al. 2002) |
|  | MLR | TTCATTGCRTAGTTWGGRTAGTT |  |  |  |

**References**

Aitken CM, Jones DM, Maguire MJ, Gray ND, Sherry A, Bowler BFJ, Ditchfield AK, Larter SR, Head IM (2013) Evidence that crude oil alkane activation proceeds by different mechanisms under sulfate-reducing and methanogenic conditions. Geochim Cosmochim Ac 109 (0):162-174. doi:10.1016/j.gca.2013.01.031

Luton PE, Wayne JM, Sharp RJ, Riley PW (2002) The *mcrA* gene as an alternative to 16S rRNA in the phylogenetic analysis of methanogen populations in landfill. Microbiology 148 (11):3521-3530

Yu Y, Lee C, Kim J, Hwang S (2005) Group‐specific primer and probe sets to detect methanogenic communities using quantitative real‐time polymerase chain reaction. Biotechnol Bioeng 89 (6):670-679. doi:10.1002/bit.20347
